# A modified double tract reconstruction with the NI method following proximal gastrectomy: a novel approach to prevent reflux and preserve nutritional status

**DOI:** 10.1007/s00464-025-12287-y

**Published:** 2025-10-23

**Authors:** Toshikatsu Tsuji, Noriyuki Inaki, Shinichi Kadoya, Jun Kinoshita, Hideki Moriyama, Daisuke Yamamoto, Hiroto Saito, Ryota Matsui, Saki Hayashi, Kengo Hayashi, Yusuke Sakimura, Kenta Doden, Hiroshi Saito

**Affiliations:** 1 Department of Gastrointestinal Surgery, Kanazawa University Hospital, 13-1 Takaramachi, Kanazawa, Ishikawa 920-8641 Japan; 2 Department of Gastroenterological Surgery, Ishikawa Prefectural Central Hospital, Ishikawa, Japan

**Keywords:** Proximal gastrectomy, Double tract reconstruction, Gastrojejunostomy, Body weight, Gastroesophageal reflux

## Abstract

**Background:**

Double-tract reconstruction is commonly performed after proximal gastrectomy to prevent gastroesophageal reflux and ensure adequate nutrition. We developed a modified double-tract reconstruction intervention, denominated the NI method, in which the anastomosis between the remnant stomach and jejunum was strategically configured to optimize food passage and suppress gastroesophageal reflux.

**Methods:**

This retrospective study included patients with upper-third gastric cancer and adenocarcinoma of the esophagogastric junction who underwent proximal gastrectomy with either conventional or NI-modified double-tract reconstruction at two institutions. Postoperative outcomes, including body weight loss and incidence of reflux esophagitis, were compared.

**Results:**

In total, 115 patients underwent proximal gastrectomy with double-tract reconstruction, including 35 with the NI method and 110 using the conventional method. No significant differences were observed in the baseline characteristics between the two groups. The incidence of reflux esophagitis was significantly lower in the NI group (0% vs. 14.3%, *P* = 0.032). Although not statistically significant, the NI group showed consistently lower weight loss rates, with approximately 2% less reduction at both 6 and 12 months after surgery.

**Conclusions:**

The NI-modified double-tract reconstruction is a safe and function-preserving technique for proximal gastrectomy. The unique gastrojejunostomy design may contribute to reduce reflux and achieve better nutritional outcomes by restoring more physiological food passage.

**Supplementary Information:**

The online version of this article contains supplementary material available 10.1007/s00464-025-12287-y.

The incidence of gastric cancer has been gradually declining in recent years, largely because of the widespread implementation of *Helicobacter pylori* eradication therapy and the decreasing prevalence of infection [1]. Nevertheless, the incidence of upper-third gastric cancer and adenocarcinoma of the esophagogastric junction (EGJ) has been increasing, particularly in East Asia, which is thought to be associated with the westernization of dietary habits and the increased prevalence of obesity and gastroesophageal reflux disease (GERD) [2, 3].

For patients diagnosed with early stage cancers located in the upper stomach or EGJ, proximal gastrectomy (PG) has been widely adopted as a function-preserving surgery offering oncological safety while preserving the gastric reservoir function [4, 5]. However, the optimal method of reconstruction following PG remains controversial, with various techniques such as esophagogastrostomy (EG), jejunal interposition (JI), and double tract reconstruction (DTR) being employed in different institutions [6–8].

DTR has gained attention for its anti-reflux benefits compared with esophagogastrostomy, as it allows partial food passage through the jejunal limb while preserving the continuity between the esophagus and the residual stomach [9]. Despite these theoretical advantages, patients may experience postoperative complications, such as reflux symptoms, delayed gastric emptying, and significant body weight loss, which can adversely affect their postoperative quality of life (QOL).

Minimally invasive approaches, such as laparoscopy and robotic surgery, have become standard in gastric cancer surgery, with attention has increasingly shifted toward optimizing postoperative function and maintaining patient-reported QOL. In this context, the refinement of reconstruction methods is warranted to better replicate physiological food flow and prevent reflux-related symptoms.

To address these challenges, we developed a modified version of DTR, referred to as the NI method, which involves technical adjustments in jejunogastrostomy to enhance physiological food passage and prevent reflux. In this study, we retrospectively evaluated the clinical efficacy of this modified reconstruction method, with a particular focus on postoperative nutritional outcomes and incidence of reflux esophagitis.

## Materials and methods

### Study design and patients

This retrospective study enrolled patients with upper-third gastric and EGJ cancers who underwent DTR following laparoscopic or robotic proximal gastrectomy between April 2010 and November 2024 at Kanazawa University Hospital and Ishikawa Prefectural Central Hospital in Japan. Patients (i) with residual gastric cancer and (ii) with concomitant malignancies in other organs Clinical and laboratory data, including medical records and images, were retrospectively collected from the hospital’s electronic patient record system. Clinical and pathological stages were classified according to the 15th edition of the Japanese Classification of Gastric Carcinomas. Patients were divided into a conventional DTR group (conventional group) and a modified DTR group (NI group).

The study design was approved by the Institutional Ethics Committee (approval number: 114334–1) of Kanazawa University and Ishikawa Prefectural Central Hospital (approval number: 2463). All procedures were conducted in accordance with the ethical standards of the committee responsible for human experimentation (institutional and national) and the Helsinki Declaration of 1964 and its later versions. An opt-out recruitment method was used for all patients who declined to participate.

### Surgical procedure

For both groups, esophagojejunostomy was performed using the overlap method following creation of the Y-limb. Two experienced surgeons performed the NI-modified DTR, whereas the conventional DTR was performed by multiple surgeons across the two institutions. The NI method was originally developed in 2016 and has since been performed exclusively by these two surgeons. All other surgeons during the study period continued to perform conventional DTR. Therefore, the allocation of patients to the NI group was based on the time of technique adoption and surgeon availability, rather than randomization, which may have introduced selection bias.

#### Conventional method

A small enterotomy was performed approximately 15 cm distal to the esophagojejunostomy on the jejunum and another enterotomy was performed on the anterior wall of the remnant stomach. Side-to-side jejunogastrostomy was performed using a linear stapler inserted toward the oral side of the jejunum.

#### NI method

**Fig. 1:**
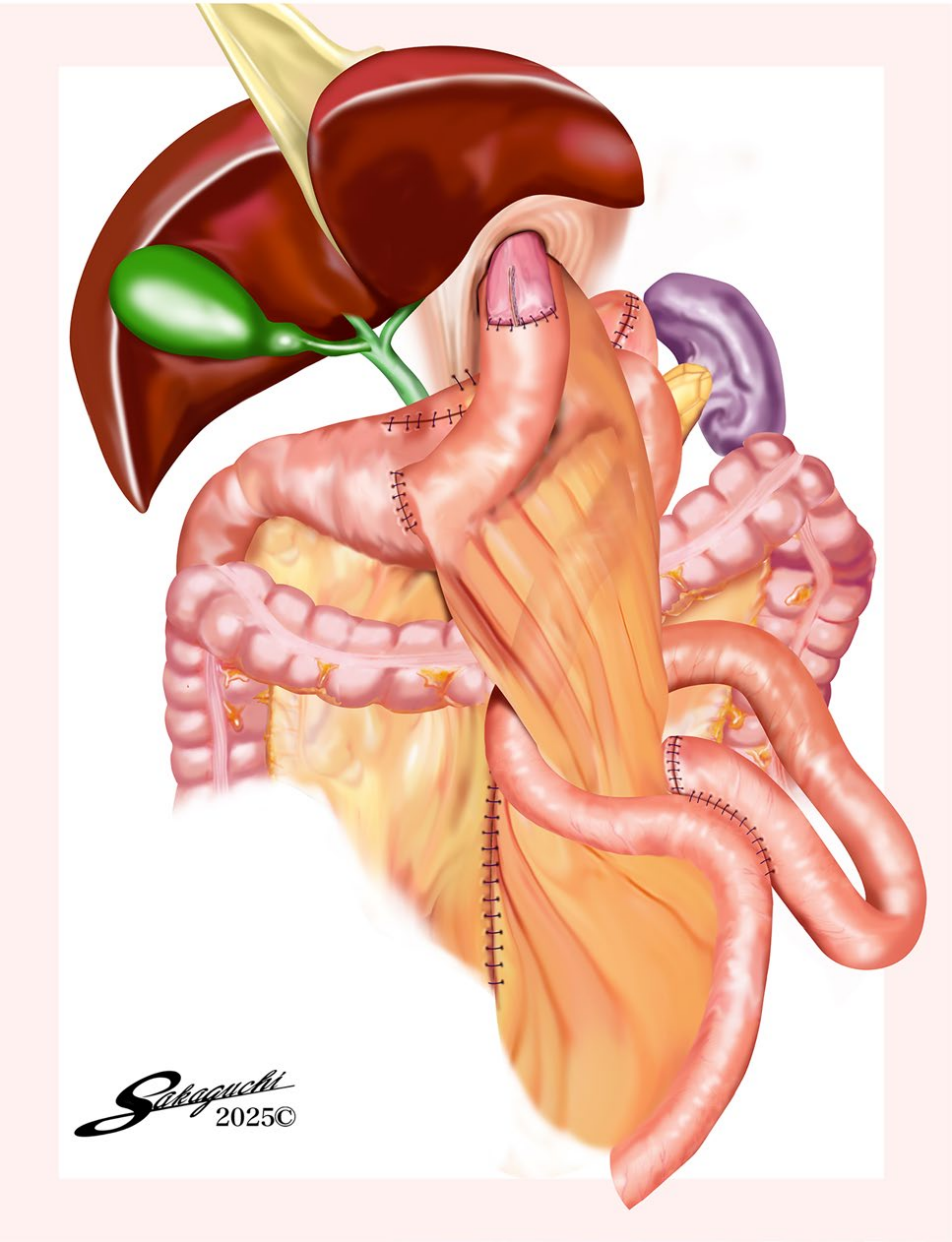
Completion schema of NI method

**Fig. 2:**
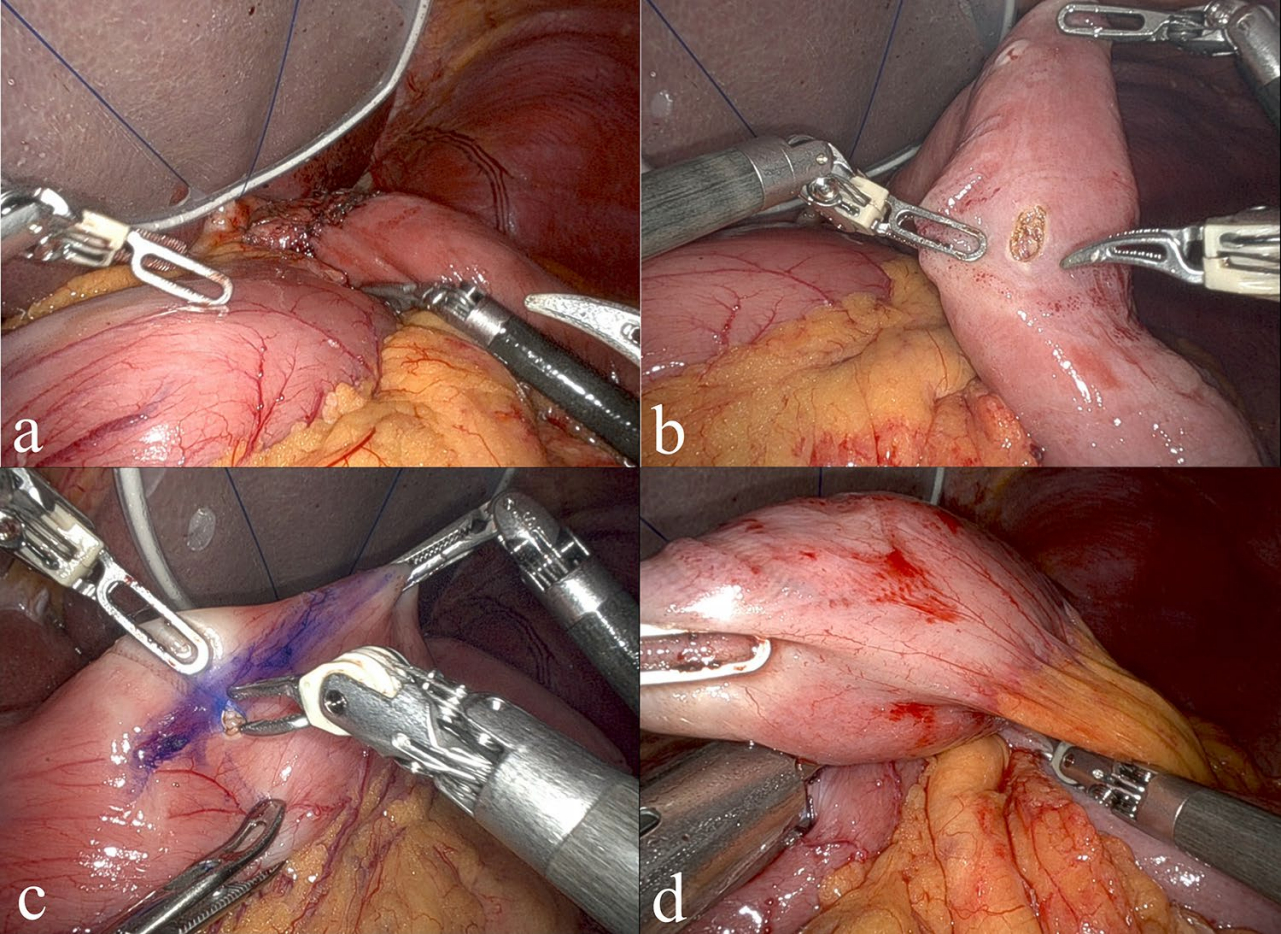
NI method. **a** The remnant stomach is positioned in a straight line dorsally to the esophagojejunostomy. **b** A small enterotomy is created 15 cm distal to the esophagojejunostomy site. **c** The anastomosis line is marked on the remnant stomach. A small enterotomy is created at the anterior wall of the lower body to antral region of the remnant stomach. **d** The elevated jejunum is bent into an N-shape and anastomosed to the remnant. stomach

The NI method is a modified technique for creating a jejunogastrostomy after proximal gastrectomy. The name “NI” comes from the shape of the reconstruction: the jejunal limb is bent into an “N” shape, while the remnant stomach is positioned straight like an “I.” These two components overlap behind the esophagojejunostomy site, forming a characteristic N-I configuration (Fig. 1). The procedure was performed by first placing the remnant stomach in a straight vertical line behind the esophagojejunostomy site (Fig. 2a). The lesser curvature of the remnant stomach was anchored to the right crus of the diaphragm using two sutures for stability. Next, a small opening was made in the jejunum, 15 cm downstream from the esophagojejunostomy (Fig. 2b). By connecting the remnant stomach to the jejunum on the anal side of this opening, we ensured a precise distance of 15 cm between the two anastomoses. This spacing is believed to reduce the risk of postoperative reflux. An opening was made in the anterior wall of the lower body to the antral region of the remnant stomach (Fig. 2c). Before creating the anastomosis, the direction of the staple line was carefully simulated and marked to optimize food flow. We assumed that by placing the anastomosis closer to the pyloric side this would help direct the food stream smoothly into the remnant stomach and mimic the effect of the gastric fundus. The cartridge of the linear stapler was inserted into the remnant stomach whereas the anvil was placed in the jejunum and oriented toward the anal side. At this point, the elevated jejunal limb was bent into an “N” shape and connected to the stomach (Fig. 2d). Before stapling, care was taken to avoid trapping the mesentery within the staple line.

This N-shaped configuration was designed to minimize the flow of food into the jejunal escape loop and maximize its passage into the remnant stomach, thereby enhancing its functional use. The common entry hole was closed using a continuous suture with a 3–0 barbed absorbable thread. In some patients, a stapler was used to save time. When using a stapler, the entry hole was temporarily closed to form a V-shaped staple line to secure closure. (Supporting information; Video).

### Outcomes

Postoperative outcomes were assessed based on clinical records and scheduled follow-up visits. Nutritional status was evaluated using body weight loss (BWL), serum albumin levels, the prognostic nutritional index (PNI), and the geriatric nutritional risk index (GNRI). Body weight was measured preoperatively and 1, 6, and 12 months postoperatively. BWL was calculated as a percentage of the preoperative weight. Serum albumin levels, and the PNI and GNRI scores were assessed preoperatively and 6 months postoperatively. The PNI was calculated as: 10 × serum albumin (g/dL) + 0.005 × total lymphocyte count (/mm^3^). The GNRI was calculated as: 14.89 × serum albumin (g/dL) + 41.7 × (body weight/ideal body weight), with the ideal body weight estimated using the Lorentz formula.

Postoperative complications within 30 days were categorized using the Clavien–Dindo classification, with only events of grade 2 or higher being evaluated. Complications included surgical site infection (SSI), anastomotic leakage, intra-abdominal abscess, pneumonia, and pancreatic fistulas, which were diagnosed based on clinical symptoms, laboratory data, or imaging studies.

GERD was evaluated at 12 months postoperatively using upper gastrointestinal endoscopy and classified according to the Los Angeles classification. In patients with reflux symptoms before this time point, an earlier endoscopic evaluation was conducted as needed. Anastomotic stricture was defined as dysphagia requiring endoscopic balloon dilation and assessed within the first year after surgery.

### Adjuvant chemotherapy

Adjuvant chemotherapy was administered in accordance with the Japanese Gastric Cancer Treatment Guidelines. Patients received either docetaxel plus S-1 (DS) or S-1 monotherapy, depending on their clinical stage and general condition. In cases of adverse events, dose reduction was performed according to the guidelines. Chemotherapy was continued for up to 1 year postoperatively, and no other treatment was administered until recurrence. In the event of recurrence, chemotherapy was initiated in accordance with the Japanese gastric cancer treatment guidelines.

### Statistical analysis

Continuous variables were expressed as medians and compared using the Mann–Whitney U test. Categorical variables were compared using the chi-squared test, as appropriate. All statistical analyses were performed using EZR (Saitama Medical Center, Jichi Medical University, Saitama, Japan) based on R (The R Foundation for Statistical Computing, Vienna, Austria) and R commander [10]. Statistical significance was defined as a *P*-value < 0.05.

## Results

In total, 115 patients were included in this study, with 80 and 35 patients in the conventional and NI groups, respectively.

### Patient characteristics

As shown in Table 1, the baseline characteristics between the two groups were not significantly different.

**Table 1:** Characteristics of patients

	Conventional group (*n* = 80)	NI group (*n* = 35)	*P* value
Age (years)			
Median, (95% CI)	69 (66.0–71.0)	69 (65.0–73.0)	0.942
Sex (%)			
Male/female	65 (81.2)/15 (18.8)	25 (77.1)/8 (22.9)	0.62
BMI (kg/m^2^)			
Median, (95% CI)	23.03 (22.0–23.66)	23.40 (21.26–24.16)	0.947
ASA-PS (%)			
0/1/2/3/4	1 (1.2)/9 (11.2)/63 (78.8)/6 (7.5)/1 (1.2)	0 (0)/1 (2.9)/32 (91.4)/2(5.7)/0 (0)	0.574
Comorbidity (%)			
CHF	4 (5.0)	2 (5.7)	> 0.999
CKD	4 (5.0)	2 (5.7)	> 0.999
COPD	5 (6.2)	5 (14.3)	0.17
DM	9 (11.2)	7 (20.0)	0.246
EGJ cancer (%)	20 (25)	12 (34.3)	0.367
Clinical stage (%)			
I/II/III/IV	66 (82.5)/9 (11.2)/4 (5)/1 (1.2)	30 (85.7)/2 (5.7)/3 (8.6)/0(0)	0.677

*CI* Confidence Interval*, BMI* Body mass index, *ASA-PS* American Society of Anesthesiologists- Physical Status, *CHF* chronic heart failure, *CKD* chronic kidney disease, *COPD* chronic obstructive pulmonary disease, *EGJ* Esophagogastric junction

### Surgical and postoperative outcomes

The surgical and postoperative outcomes are summarized in Table 2. No significant differences between the two groups were observed in terms of operative time, blood loss, extent of lymph node dissection, postoperative complications (including SSI, anastomotic leakage, intra-abdominal abscess, pneumonia, or pancreatic fistula), pathological stage, postoperative hospital stay, incidence of adjuvant chemotherapy, or anastomotic stenosis. Notably, no cases of GERD were observed in the NI group during the 1-year postoperative follow-up, and the incidence was significantly lower than that in the conventional group (*P* = 0.032).

**Table 2:** Operative and postoperative outcomes

	Conventional group (*n* = 80)	NI group (*n* = 35)	*P* value
Operative time (min)			
Median, (95% CI)	281.0 (264.0–295.0)	254.0 (235.0–333.0)	0.32
Blood loss (g)			
Median, (95% CI)	12.5 (10.0–20.0)	10.0 (7.0–13.0)	0.067
Lymph node dissection (%)			
D1/D1+/D2	19 (23.8)/57 (71.2)/4 (5)	7 (20)/26 (74.3)/2 (5.7)	0.936
Postoperative complication			
Total complications, > CD-2 (%)	19 (23.8)	6 (17.1)	0.474
Incisional SSI	2 (2.5)	0 (0.0)	> 0.999
Anastomotic leakage	6 (7.5)	3 (8.6)	> 0.999
Pneumonia	3 (3.8)	0 (0.0)	0.552
Intra-abdominal abscess	9 (11.2)	3 (8.6)	> 0.999
Pancreatic fistula	1 (1.2)	0 (0.0)	> 0.999
Pathological stage (%)			
I/II/III/IV	63 (78.8)/11 (13.8)/5 (6.2)/1 (1.2)	25 (71.4)/5 (14.3)/5 (14.3)/0 (0)	0.528
Postoperative hospital stay (days)			
Median, (95% CI)	15.0 (13.0–17.0)	14.0 (12.0–15.0)	0.17
Adjuvant chemotherapy (%)	12 (15.0)	9 (25.7)	0.195
Anastomotic stenosis (%)	4 (5.0)	0 (0.0)	0.312
GERD (%)	11 (14.3)	0 (0.0)	0.032

*CI* Confidence Interval*, CD* Clavien Dindo classification, *SSI* surgical site infection, *GERD* gastroesophageal reflux disease

### Nutritional outcomes

As shown in Table 3, the nutritional status was evaluated. No significant differences between the two groups were observed in terms of serum albumin levels, or the PNI and GNRI scores. Although none of the differences were statistically significant (*P* = 0.07 at 1 month, *P* = 0.275 at 6 months, and *P* = 0.075 at 12 months), the NI group consistently showed approximately 2% lower BWL rates at both 6 and 12 months.

**Table 3:** Evaluation of nutritional parameters

	Conventional group (*n* = 80)	NI group (*n* = 35)	*P* value
Postoperative BWL (%), median (95% CI)			
1 month	7.27 (6.75–8.18)	6.76 (4.38–8.37)	0.07
6 months	14.46 (11.68–17.59)	12.32 (10.5–14.58)	0.257
1 year	15.68 (14.23–18.73)	13.29 (10.04–16.03)	0.075
Serum albumin (g/dL), median (95% CI)			
Before surgery	4.20 (4.10–4.40)	4.20 (3.90–4.40)	0.55
After surgery	3.95 (3.80–4.10)	4.00 (3.90–4.00)	0.931
PNI, median (95% CI)			
Before surgery	49.00 (47.92–51.96)	49.84 (47.80–52.30)	0.733
After surgery	47.38 (44.88–49.45)	48.35 (45.30–50.60)	0.744
GNRI, median (95% CI)			
Before surgery	111.30 (108.8–114.2)	112.70 (106.0–115.66)	0.726
After surgery	100.55 (98.15–103.05)	103.35 (98.45–106.55)	0.559

*CI* Confidence Interval*, BWL* body weight loss, *PNI* prognostic nutritional index, *GNRI* geriatric nutritional risk index

## Discussion

**Fig. 3:**
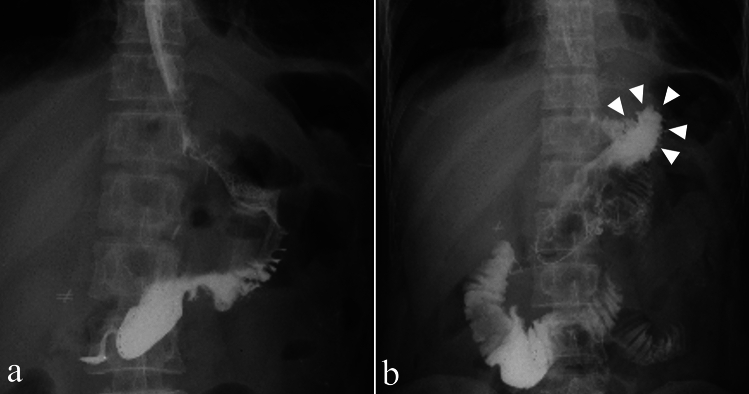
Postoperative day 3 fluoroscopic images using Gastrografin following NI-modified DTR. **a** Upright position. Gastrografin passes smoothly into the remnant stomach without delay. **b** Supine position. Gastrografin is retained in the neofundic area, with no evidence of reflux into the esophagus (arrowhead)

The present study demonstrated that our modified DTR, the NI method, was not associated with the incidence of reflux esophagitis and achieved consistently lower postoperative BWL rates than the conventional DTR. Although the differences in weight loss did not reach statistical significance, the NI group maintained an approximately 2% lower weight loss at both 6 and 12 months postoperatively. While this degree of difference may appear modest, it may still have clinical relevance in the long-term recovery and nutritional maintenance of patients, especially when accumulated over time or in more vulnerable populations. These findings suggest that the NI method may offer potential advantages in reducing postoperative gastroesophageal reflux and supporting nutritional preservation. We believe that these favorable outcomes are due to the increased physiological passage of food achieved by the specific anastomosis between the remnant stomach and jejunum. Postoperative contrast studies supported this finding, revealed a smooth flow of contrast into the remnant stomach and no reflux into the esophagus, even in the supine position (Fig. 3).

Several reconstruction methods have been reported following proximal gastrectomy, including EG, JI, and DTR. EG is technically simple and time-saving but often results in postoperative reflux esophagitis unless an anti-reflux procedure is incorporated. To address this limitation, anti-reflux procedures such as the double-flap technique (DFT) and side-overlap with fundoplication (SOFY) have been introduced. DFT, first proposed by Kamikawa et al. and later refined, involves creating a seromuscular double flap on the anterior wall of the remnant stomach and has demonstrated favorable reflux control [11]. More recently, Yamashita et al. described the SOFY technique, which also showed efficacy in preventing reflux while simplifying the anastomosis [12]. JI involves inserting a jejunal segment between the esophagus and remnant stomach to act as a reflux barrier. Although this method can preserve the gastric reservoir function and minimize reflux, it requires three anastomoses and a longer operative time, potentially increasing the risk of complications [13]. DTR provides a balance between reflux control and nutritional preservation by creating dual pathways for food passage. However, DTR can be technically challenging in patients with extensive intra-abdominal adhesions, particularly those involving the small intestine. Importantly, no randomized controlled trials have directly compared these reconstruction techniques. Thus, definitive conclusions regarding their superiority remain elusive. In the present study, although surgeries were conducted at two institutions, the NI-modified DTR procedures were performed exclusively by two experienced surgeons using a standardized technique.

In contrast, conventional DTR procedures were performed by multiple surgeons, potentially introducing variability. Nonetheless, the consistent and favorable outcomes observed with the NI method, particularly the reduced incidence of reflux and better preservation of body weight, suggest that its unique gastrojejunostomy configuration may contribute to increased physiological reconstruction and improved functional recovery. With the advancement of minimally invasive surgical techniques, such as laparoscopic and robot-assisted procedures, improvements in surgical safety and precision have been achieved. However, technical excellence alone does not ensure optimal patient-centered outcomes. Postoperative QOL has emerged as a critical factor for evaluating the success of gastrectomy, particularly in function-preserving surgeries such as PG. The Post-Gastrectomy Syndrome Assessment Scale (PGSAS)-45 was developed to comprehensively evaluate the QOL of patients after gastrectomy [14]. Using this tool, Takiguchi et al. demonstrated that patients who underwent PG had significantly better outcomes in terms of body weight maintenance, meal-related distress, and overall satisfaction than those undergoing total gastrectomy [15]. More recently, Ikeda et al. reported that among PG reconstruction techniques, DTR was associated with superior QOL scores in domains such as reflux, ingestion, and physical well-being compared with EG, particularly in patients with smaller remnant stomachs [16]. In the present study, we did not directly assess postoperative quality of life using validated instruments such as PGSAS-45, due to the retrospective nature of the study design. However, the favorable outcomes observed in terms of reflux prevention and nutritional parameters may suggest a potential QOL benefit of the NI method. Future prospective studies incorporating QOL assessment tools are warranted to further evaluate this technique’s impact on patient-centered outcomes.

DTR has thus been recognized as a functionally advantageous and safe reconstructive option following PG, offering both effective reflux prevention and long-term nutritional benefits [17, 18]. In the present study, the NI-modified DTR, in which the anastomosis between the remnant stomach and jejunum is strategically modified, showed further potential benefits with no cases of reflux esophagitis and a sustained reduction in weight loss of approximately 2%, although the difference was not statistically significant. Notably, this was accomplished by altering only the gastrojejunostomy configuration, without additional anastomoses or prolonging the operating time. These results support the notion that even minor technical refinements in reconstruction can have a meaningful impact on the postoperative outcome and QOL. Therefore, the NI method may serve as a promising patient-centered advancement in reconstructive strategies following PG. However, our study only included a follow-up period of 12 months. While this timeframe is commonly used in studies evaluating short- to mid-term outcomes, it may not fully capture the potential for late-onset reflux symptoms or delayed nutritional decline. Long-term follow-up will be essential to determine the durability of the observed benefits and to evaluate any delayed complications or changes in functional outcomes.

This study had several limitations that should be acknowledged. First, the sample size was relatively small, which may have limited the statistical power and generalizability of the findings. Second, the retrospective nature of this study introduces a potential selection bias. Although the NI group included consecutive patients who underwent the modified technique during a defined period at two centers, selection bias may still exist due to surgeon preference, patient background, or institutional practices. In addition, unmeasured confounding factors such as preoperative nutritional status, postoperative care differences, or patient adherence to dietary guidance could not be fully controlled for. Future prospective studies with standardized protocols and randomization will be necessary to minimize such biases. Third, we did not directly compare the NI method with other reconstruction techniques, such as EG or JI; therefore, it is not appropriate to draw definitive conclusions regarding the superiority of the NI method over all existing techniques. Although our findings are encouraging, further research is required before recommending its widespread adoption.

In conclusion, the NI method appears to be a safe and effective modification of conventional DTR following PG, offering promising results in terms of reflux prevention and nutritional preservation. Prospective multicenter studies involving larger cohorts and standardized QOL assessments are necessary to validate these findings and further clarify the optimal reconstruction strategy.

## Supplementary Information

Below is the link to the electronic supplementary material.

**Supporting information; Video:** This video demonstrates that a modified version of DTR, referred to as the NI method. (AVCHD Video 4,13,842 kb)
